# Lung organoids in COPD: recent advances and future prospects

**DOI:** 10.1186/s12931-025-03138-4

**Published:** 2025-02-28

**Authors:** Yajie Huo, Shengyang He, Yan Chen

**Affiliations:** 1 Department of Pulmonary and Critical Care Medicine, The Second Xiangya Hospital of Central South University, Changsha, Hunan China; 2 Research Unit of Respiratory Disease, Central South University, Changsha, Hunan China; 3 Clinical Medical Research Center for Pulmonary and Critical Care Medicine in Hunan Province, Changsha, Hunan China; 4 Diagnosis and Treatment Center of Respiratory Disease in Hunan Province, Changsha, Hunan China

**Keywords:** Lung organoids, Stem cells, COPD, Lung progenitors

## Abstract

Chronic obstructive pulmonary disease (COPD) is a chronic inflammatory airway disease that is characterized by progressive airflow limitation, a high prevalence, and a high mortality rate. However, the specific mechanisms remain unclear, partly due to the lack of robust data from in vitro experimental models and animal models that do not adequately represent the structure and pathophysiology of the human lung. The recent advancement of lung organoid culture systems has facilitated new avenues for the investigation of COPD. Lung organoids are in vitro models derived from adult stem cells, human pluripotent stem cells, or embryonic stem cells, established through three-dimensional culture. They exhibit a high degree of homology and genetic consistency with human tissues and can better mimic human lungs in terms of function and structure compared to other traditional models. This review will summarise the generation process of lung organoids from different cell sources and their application in COPD research, and provide suggestions for future research directions.

## Introduction

Chronic obstructive pulmonary disease (COPD) is a common respiratory disease with high morbidity and mortality, characterized by irreversible airflow limitation [1]. Many animal models have been developed and widely used to study the pathogenesis of COPD, and traditional animal models have significant research value in certain specific contexts. However, due to species and genetic differences between humans and other animals, existing animal models have certain limitations and cannot fully reflect the physiological and pathological characteristics of humans [2]. In addition, these models cannot adequately mimic the cellular complexity of the human lung [3]. Therefore, there is an urgent need to use recently developed models to better understand the pathophysiology of COPD-related lung disease in vivo. In this context, lung organoids, with their ability to self-renew and differentiate, have attracted the attention of the research community to investigate the mechanisms and potential treatments of COPD [4, 5].

Lung organoids, which have structural characteristics and functions similar to certain real human organs and tissues, are developed from either embryonic stem cells (ESCs), induced pluripotent stem cells (iPSCs) or adult stem cells (ASCs) and cultured to generate three-dimensional cultures of tissues such as alveoli, airways and lung buds [6, 7]. The culture process involves two key stages. First, prior to stem cell differentiation, key signalling pathways that control developmental patterns are carefully modulated, either activated or inhibited, to shape the correct regional identities. The culture then progresses with the incorporation of a three-dimensional extracellular matrix (or microgel) that promotes a more complex environment. Media formulations are created by adapting established two-dimensional culture techniques or by taking inspiration from mouse development [8]. In general, the use of different stem cell sources and appropriate culture conditions allows directed differentiation and long-term maintenance of lung organoids [9]. Lung organoids outline cell-cell and cell-niche interactions in development, homeostasis and disease, and can be extended to high-throughput screening for small molecules that determine cell fate. In addition, organoids derived from human cells have great advantages for studying human epithelial stem cell biology and modelling human disease [10]. As a result, lung organoids have become an indispensable tool for in vitro modelling of organ development, regeneration and disease.

In this review, we focus on the origin of lung organoids and their potential in the study of COPD pathogenesis, drug screening and personalized treatment. Lung organoids, derived from human stem cells, better replicate human lung features and disease mechanisms than animal or 2D models. This provides an important experimental platform for understanding COPD mechanisms and developing novel therapeutic strategies. In addition, we summarize the limitations and future directions of lung organoids in COPD research, providing new perspectives and insights to advance research in this field.

**Fig. 1:**
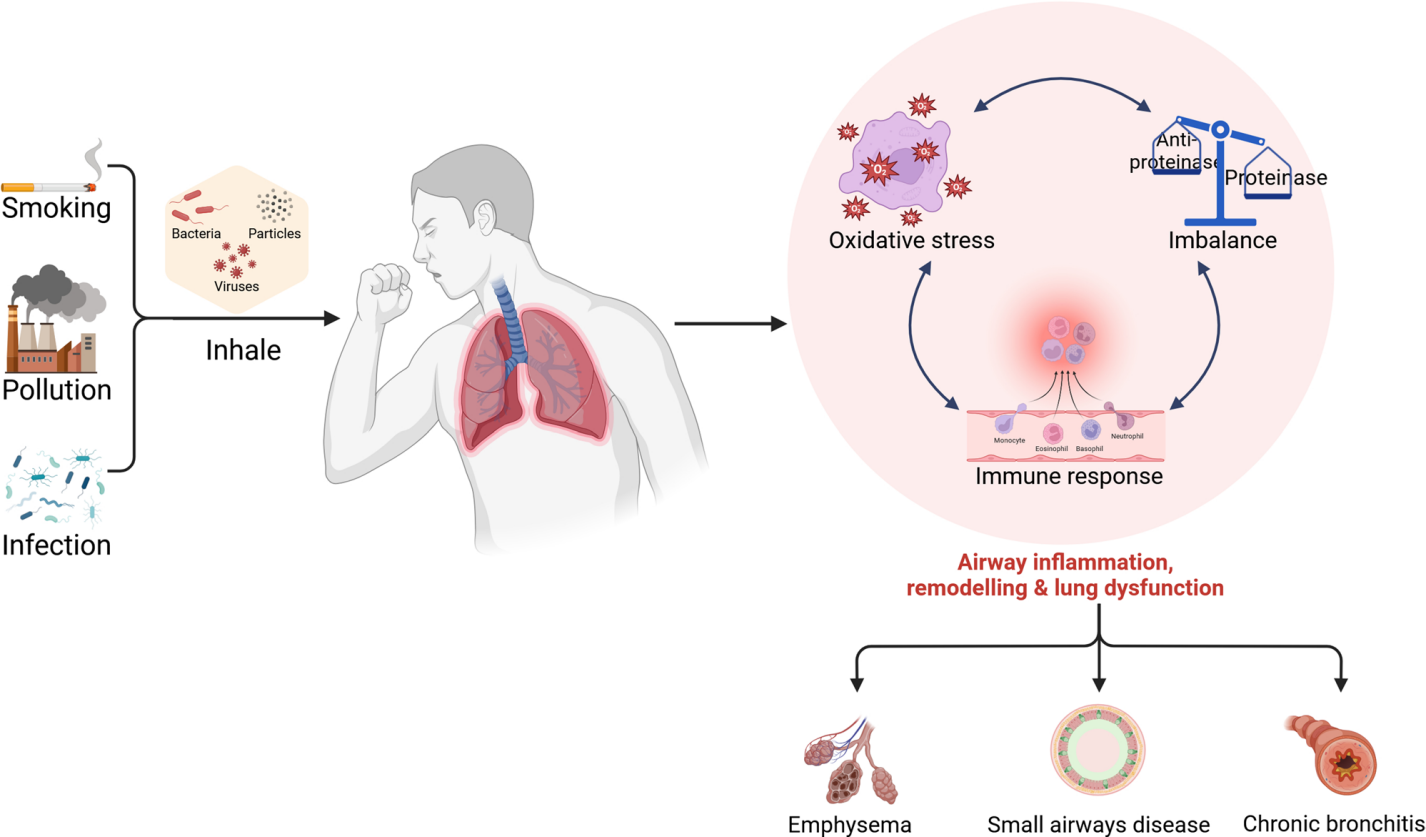
The pathogenesis of COPD. The primary etiological factors of COPD include cigarette smoke, environmental pollution, occupational dust and chemical exposure, and infections. The pathogenesis of COPD is characterized by chronic inflammation of the airways, lung parenchyma and pulmonary blood vessels, a disruption in the balance between proteinase and antiprotease, and an increase in oxidative stress. These factors act in concert to precipitate pathological alterations in emphysema, chronic bronchitis and small airway remodeling

## Chronic obstructive pulmonary disease (COPD)

COPD is the third leading cause of morbidity and mortality worldwide. A preventable disease with effective treatment options, COPD is characterized by persistent airflow limitation and acute exacerbation of the chronic inflammatory response of the airways and lung tissues to inhaled toxic substances and gases [11]. The pathogenesis of COPD is poorly understood and the mainstream view is that the onset of COPD is the result of an interaction between the environment and personal genetic characteristics. In western countries, long-term smoking is the main cause of COPD, while in developing countries factors such as the burning of biomass fuels for cooking and heating may also play a significant role. However, smoking remains a major contributor to COPD in both contexts [12]. Other risk factors for COPD include occupational exposure to air pollutants such as coal ash and dust, airway hypersensitivity caused by exposure to allergens, and certain family predispositions [13].

The development of COPD is driven by mechanisms such as oxidative stress, protease-antiprotease imbalance and immune dysregulation [14]. Oxidative stress, triggered by cigarette smoke and pollutants, increases reactive oxygen species in the lungs, damaging cells and promoting inflammation leading to emphysema [15]. Excessive protease activity, particularly matrix metalloproteinases, degrades the extracellular matrix (ECM), destabilizing lung structure [16]. Immune dysregulation further exacerbates COPD by promoting inflammatory cell infiltration, exacerbating airway inflammation [17]. These mechanisms interact with each other, ultimately leading to emphysema, chronic bronchitis, and small airway remodeling. (Fig. 1) [18]. Furthermore, during the pathogenetic process of COPD, bronchial epithelial cells and alveolar cells are the first barriers of the airways against external stimuli. Once damaged, they are not only challenged themselves but also affect the surrounding microenvironment. These dynamic interactions could subsequently trigger persistent chronic inflammatory responses and extensive remodelling of lung tissue structure, ultimately compromising lung function and leading to the progression of COPD [19].

Despite some progress, the molecular mechanisms of COPD remain poorly understood, and effective therapeutic strategies to halt or reverse disease progression have not been developed. Traditional 2D cell culture models lack the complexity of multicellular layers and tissue structure, making it difficult to accurately replicate the COPD microenvironment and capture the unique cellular interactions and disease progression of the human lung. In contrast, organoid models can more faithfully simulate the cellular diversity and microenvironment of the human lung by forming three-dimensional cellular structures. They not only mimic the interactions between key cell types involved in COPD, such as airway epithelial cells, immune cells and fibroblasts, but also reflect processes such as cellular migration, inflammatory responses and tissue remodeling. These advantages make organoids a powerful tool for studying the pathogenesis of COPD and advancing therapeutic strategies. Therefore, a review of organoid research methods can provide researchers with a deeper understanding of this emerging technology, fostering further progress in the field.

## Lung and lung organoids

An understanding of the structure of the lung is beneficial in order to facilitate comprehension of the use of lung organoids in disease. The lung is a highly complex organ comprising a highly branched system of tubes that facilitate the transportation of air to the alveoli, where gas exchange occurs. The adult lung is subdivided into a proximal and a distal region. The proximal region, which encompasses the trachea and bronchioles, constitutes the conduction zone (airway). In this zone, the epithelial cells of the upper airway are responsible for the removal of airborne microbes and suspended particles. The luminal surface of the airway is lined with ciliated pseudostratified columnar epithelium, containing goblet cells, ciliated cells, pulmonary neuroendocrine cells (PNECs) and basal cells, with basal cells serving as the stem or progenitor cells of the respiratory epithelial cells [20]. As the degree of branching in the airway tree increases, the epithelium undergoes a gradual transition from pseudolamellar to simple cubic, with the dominant cells becoming non-ciliated, but PNECs remain present in these regions. Club cells are a type of secretory cell found in the bronchiolar epithelium. They contribute to the production of non-mucus-secreting proteins in the extracellular lining fluid. The distal region of the lung, which is responsible for gas exchange, consists of two main types of alveolar epithelium: type 1 alveolar epithelium (AT1), type 2 alveolar epithelium (AT2), and the associated vascular system [21].

Furthermore, despite the lung’s reputation as a highly static tissue with low cell renewal, it demonstrates a robust response following injury. As a result of prolonged exposure to airborne irritants, including cigarette smoke, pollutants, and viruses, the lungs have developed a diverse range of reparative mechanisms. The activation of regional stem/progenitor cells is contingent upon the type and severity of the injury in question [22]. These include airway basal cells, which are responsible for the production of all airway epithelial cells [23]; club cells, which are capable of differentiating into ciliated cells [24]; and AT2s [25]. Recently, there has been an increase in evidence suggesting that distal airway stem/progenitor cells, including bronchiolar alveolar stem cells (BASCs) [26], co-express AT2 and club cell markers that contribute to both airway and alveolar repair. This provides further insight into the understanding of lung epithelial stem cells.

**Fig. 2:**
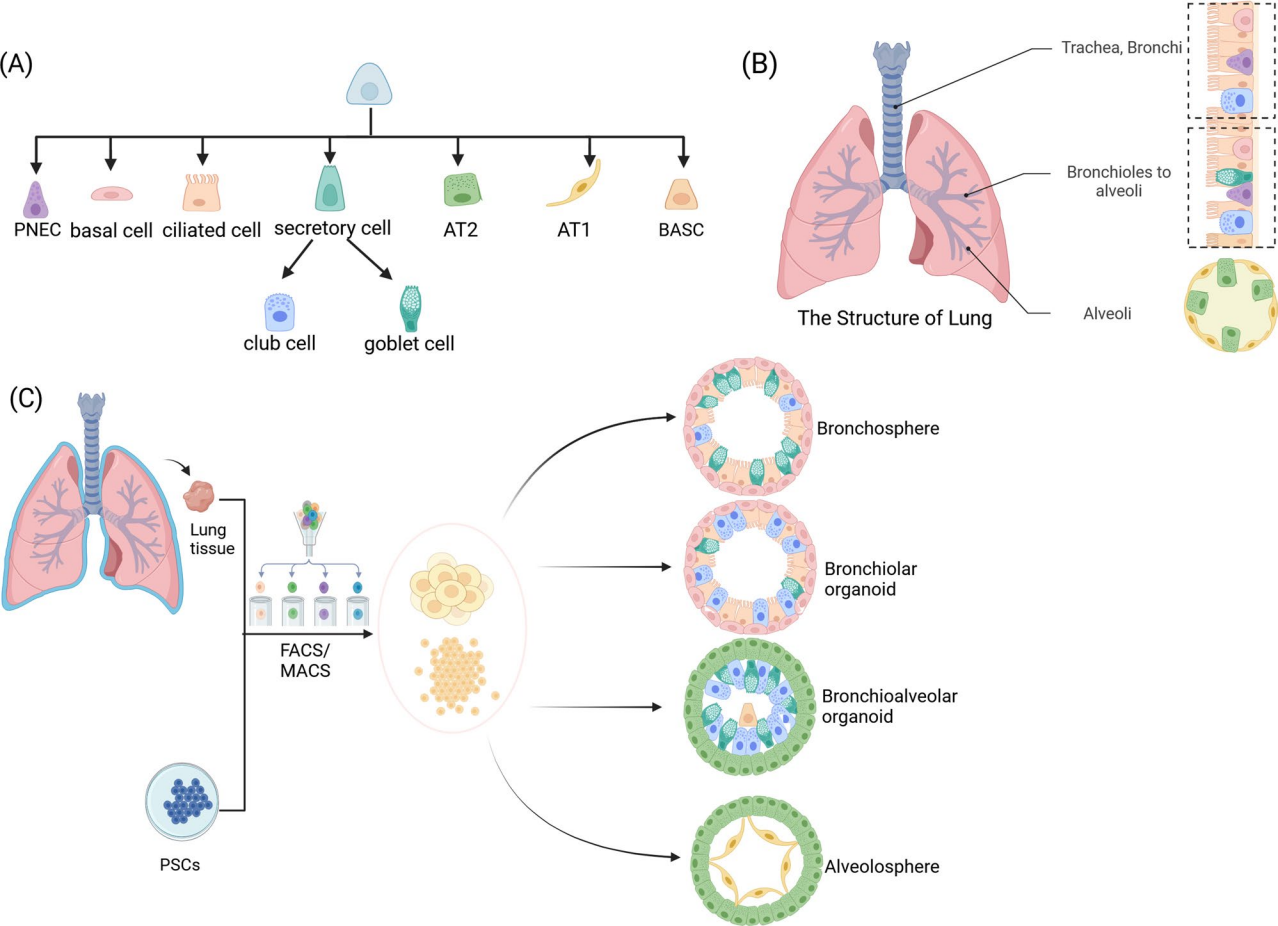
The structure of lung and lung organoid. (**A**) The cells that constitute the lungs and lung organoids. (**B**) The adult lung is subdivided into a proximal and a distal region. The proximal region, comprising the trachea and bronchioles, constitutes the conduction zone (airway). The surface of the airway lumen is lined with goblet cells, ciliated cells, and basal cells. As the degree of branching increased, the predominant cell type underwent a transition from ciliated to non-ciliated. Club cells are a type of secretory cell found in the bronchiolar epithelium. The distal region, which is responsible for gas exchange (alveoli), is composed of AT1 and AT2 cells. (**C**) Lung organoids are derived from lung stem cells that have the capacity to differentiate. They can be classified into four main categories, namely alveolosphere, bronchiolar organoids, bronchioalveolar organoids and bronchosphere, according to their structural characteristics

Lung organoids are three-dimensional, self-assembled cellular structures.

(Fig. 2) comprising a variety of cell types that are specific to the organ in question and perform the functions associated with that organ [27]. Lung organoids are derived from lung stem cells with the capacity for differentiation. They can be classified into four main categories, namely alveolosphere, bronchiolar organoids, bronchioalveolar organoids and bronchosphere/tracheosphere, based on their structural characteristics [28]. Bronchial organoids exhibit a structure and cellular composition analogous to that of smaller conducting airways. These organoids are characterized by the presence of basal cells, ciliated cells, and mucus-producing cells. In contrast, alveolar organoids are structured in a manner analogous to alveoli, comprising predominantly AT1 and AT2 cells. Given that lung organoids closely resemble the physiological characteristics and genetic diversity observed in the lung, they have been identified as invaluable resources for investigating hitherto unknown pathways, including lung development, disease pathophysiology and therapeutic biology [10]. The following section will present the methodology employed in the formation of lung organoids derived from various stem cells.

## Main sources of lung organoids

### Organoids from airway basal cells

In the proximal airway, basal cells are situated in close proximity to the basal layer of the epithelium, collectively constituting the lung stem cell population. In mice, the trachea contains a substantial number of basal cells, with some also present in the proximal airway. In humans, basal cells are present in the trachea, bronchus, and bronchioles. Basal cells possess the capacity for proliferation, self-renewal and differentiation into ciliated, goblet and club cells in vivo [29]. Lung organoids derived from basal cells are designated tracheospheres (originating in the trachea) or bronchospheres (originating in the bronchus) [23]. Basal cells express a number of genes, including those encoding p63, cytokeratin 5, integrin α6, podoplanin and the nerve growth factor receptor (NGFR) [30]. In accordance with standard conditions, these organoids should contain TRP63 + KRT5 + basal cells, ACT + FOXJ 1 + ciliated cells and secretory goblet cells (MUC5AC+, MUC5B+) [31, 32]. Basal cells are typically cultivated on Matrigel or in transwell lower attachment plates. The culture media typically comprises supplements such as epidermal growth factor, bovine pituitary extract, insulin transferrin, selenium, cholera toxin, and retinoic acid [33]. Tracheospheres and bronchospheres have been employed as screening tools for the identification of secretory factors that influence the self-renewal and differentiation of basal cells. Organoid culture has identified a number of factors that regulate the fate of basal cells, including interleukin-13 (IL-13) and interleukin-17 A (IL-17 A). These factors can directly affect basal-derived lung organoids, induce goblet cell generation, damage ciliated cells, and mimic goblet cell metaplasia (GCM) phenotype [34]. GCM is a key feature common to many airway diseases, including asthma and COPD. These findings provide a theoretical basis for the development of cytokine-specific therapy and stratification of patients based on specific cytokine levels [35]. Consequently, basal-cell-derived organoids have been employed to screen secreted proteins, small molecules, and drugs [36].

### Organoids from airway secretory cells

Secretory cells are defined as the columnar, non-ciliated, non-neuroendocrine cells present in the airway epithelium of the lung, which are more abundant in terminal bronchioles [37]. The two main types of secretory cells are club cells and goblet cells. The primary function of club cells is to safeguard the bronchiolar epithelium through the synthesis of secreted globin (SCGB1A1, SCGB3A2) and SPLUNC1 proteins. In contrast, goblet cells are responsible for the synthesis of mucins, such as MUC5AC and MUC5B, which serve to protect the lung lining [24]. In organoid culture, club cells, when co-cultured with Lgr6pos or Lgr5pos mesenchymal cells, demonstrated the formation of bronchiolar, alveolar, or bronchiolar alveolar colonies [38]. Two distinct methodologies have been employed to isolate secretory club cells. The first method employs fluorescence-activated cell sorting (FACS) to isolate cells based on specific cell markers. The second method utilizes the Scgb1a1-CreER knockin for lineage tracking [24]. The use of secretory-derived organoids represents a valuable platform for the investigation of the individual impacts of cytokines and growth factors on the proliferation and differentiation of secretory cells. Moreover, they enable the identification of particular subpopulations of club cells that display enhanced regenerative capabilities. There is substantial evidence that club cells constitute a heterogeneous population that displays considerable phenotypic plasticity in response to agents that damage either the airway or alveolar epithelium [39]. For example, lineage-tracing studies conducted following damage to the alveolar region by the chemotherapeutic drug bleomycin have demonstrated that Scgb1a1 + cells in the distal bronchioles proliferate and give rise to progeny in the alveoli with characteristics of AT2s and AT1s [25]. Nevertheless, the majority of research exploring the development of secretory cells in organoids has been conducted using mouse models. It remains uncertain whether human club cells possess the capacity to function as stem or progenitor cells.

### Organoids from airway bronchioalveolar stem cell (BASC)

It has been demonstrated that the presence of distal airway stem/progenitor cells has the potential for alveolar and airway differentiation [40]. BASCs situated at the interface between the bronchoalveolar duct and the distal lung were identified as the inaugural stem cell population in the distal lung. BASCs have been observed to co-express markers characteristic of both club cells and AT2s [26]. Following naphthalene-mediated bronchiolar injury, they produce rod and ciliary cells. Similarly, following bleomycin-induced alveolar injury, they produce AT2s and AT1s. BASC has the capacity to produce alveolar, bronchiolar, and bronchoalveolar organoids through a co-culture process with lung endothelial cells, which rely on endothelial thromboprotein-1 [40]. It is noteworthy that the bronchoalveolar organoids included AT2, club, ciliated, and goblet cells, but no AT1 cells. Furthermore, the co-culture of BASCs with resident mesenchymal cells has been demonstrated to yield complex bronchoalveolar pulmonary organoids (BALOs). These organoids may form a tubular airway-like region comprising basal cells, secretory cells, and ciliated cells, while the distal alveolar region contains differentiated AT1 and AT2 cells [41]. At present, the prevailing methodology for the separation of BASC employs FACS, with the identification of cells based on their expression of epithelial cell adhesion molecule (EpCAM) and stem cell antigen 1 (Sca1). New techniques will enable the purification of these cells on the basis of co-expression of genes encoding Scgb1a1 and Sftpc, thus facilitating the identification of additional markers for the distinction of BASC from other club cells.

### Organoids from airway alveolar epithelial type 2(AT2)

Two types of alveolar epithelial cells have been identified: the AT2 cell, which has the capacity to produce alveolar surface active substances (such as Sftpc and Sftpb), thereby reducing alveolar surface tension and preventing alveolar collapse; and the AT1 cell, which covers the majority of the alveolar surface area and performs a gas exchange function [42]. AT2-derived organoids are referred to as alveolospheres, and human AT2 cells can be isolated specifically by monoclonal antibody HTII280 via FACS or magnetic bead sorting (MACS) [43]. The first description of organoids derived from mouse and human AT2 cells was provided by Barkauskas in 2013 [25]. In this organoid model, the support provided by mesenchymal/fibroblast/endothelial cells in Matrigel enables AT2 cells to grow into alveolar spheres comprising AT2s on the exterior and AT1s within the lumen [25]. Subsequently, a number of organoid models have been developed utilizing AT2 subsets and mesenchymal cell subpopulations, with the objective of investigating interactions within the alveolar stem cell niche [44, 45]. The alveolosphere has been employed extensively in investigations into the regulation of AT2 to AT1 differentiation [46], AT2-fibroblast crosstalk, and AT2-macrophage crosstalk [47]. Recently, alveolospheres have been employed to investigate the impact of BMP signalling on AT2 proliferation. It has been demonstrated that BMP activation results in a reduction in AT2 proliferation and an increase in AT1 differentiation, whereas its inhibition stimulates AT2 self-renewal. These findings indicate that BMP regulation is of paramount importance for alveolar formation and maintenance [48]. The primary challenge in investigating the genetic functions of AT2 cells using organoids is the instability of their propagation in Matrigel. This impedes progress in understanding their roles in lung regeneration, gas exchange, immune defense, and so forth. Potential solutions include optimizing culture conditions and refining cell isolation and purification techniques.

### Organoids from human pluripotent stem cells (hPSCs)

Pluripotent stem cells, including ESCs and iPSCs, are induced to differentiate in in a specific organ-related direction after various developmental stages [4]. The development of lung organoids derived from human pluripotent stem cells (hPSCs) represents a significant advancement in the field of in vitro lung research, offering a promising platform for modelling the intricate human lung. Organoids created using hPSCs have a high degree of applicability and show great promise in simulating a range of genetic diseases, evaluating the underlying pathophysiological causes, studying the effects of customized treatments, and understanding the processes involved in lung development. Ultimately, they may offer a potential therapeutic avenue for lung repair and regeneration.

The addition of various cell media, cytokines and small molecular compounds enabled the generation of endoderm, then foregut endoderm, ventral foregut endoderm cell (VAFEC) and finally NKX 2.1 + lung progenitor cells from mouse and iPSC [4, 49, 50]. The co-culture of a human iPSC cell line and human fetal lung mesenchymal cells demonstrated that carboxypeptidase M positive (CPM+) surface markers could be employed for the isolation of pulmonary progenitor cells from VAFEC [51]. In 2015, Dye et al. reported that the culture of human lung organoids (HLOs) derived from human pluripotent stem cells. The tissue structure of the organoid is comparable to that of the lung, exhibiting both proximal airway and distal airway epithelial structures. However, it also displays immature alveolar-like regions, reminiscent of the fetal lung [52]; In 2017, Chen et al. employed hPSCs to generate lung bud organoids (LBOs). These LBOs serve as three-dimensional human lung models, exhibiting the presence of pulmonary endoderm and mesoderm. The organoid was cultivated in a three-dimensional Matrigel matrix and xenotransplantation was performed, resulting in the formation of an airway with branching and an early alveolar structure [53]. Presently, the LBO model is primarily employed to investigate the pathophysiological alterations associated with pulmonary disorders, including respiratory infection and pulmonary fibrosis [4]. In 2019, Miller et al. successfully obtained lung bud organoids and lung organoids by culturing hPSCs in three distinct stages [4]. In 2020, Dye et al. demonstrated that biomaterial scaffolds can influence the size and formation of airways in analogous organs and enhance the transplantation of HLO [54]. In 2021, Leibel et al. employed iPSC to generate three-dimensional whole lung organoids (WLO). WLO comprise distal and proximal epithelial lung cells as well as mesenchymal cells, which are employed to model disease and developmental biology [55]. WLO can be used to study signal transduction in human lung epithelium and mesenchyma, thereby simulating the impact of genetic mutations on human lung cell functionality and development. In 2022, Peng et al. described a method for co-culture of iPSC and an artificial basement membrane (ABM) to produce alveolar epithelium. The method results in the production of alveolar epithelium with highly specific protein expression and unique tight junctions. Furthermore, the establishment of the gas-blood barrier can be achieved through the co-culture of endothelial cells [56].

## Application of Lung Organoid in COPD

### Lung organoids for disease mechanism in COPD

**Fig. 3:**
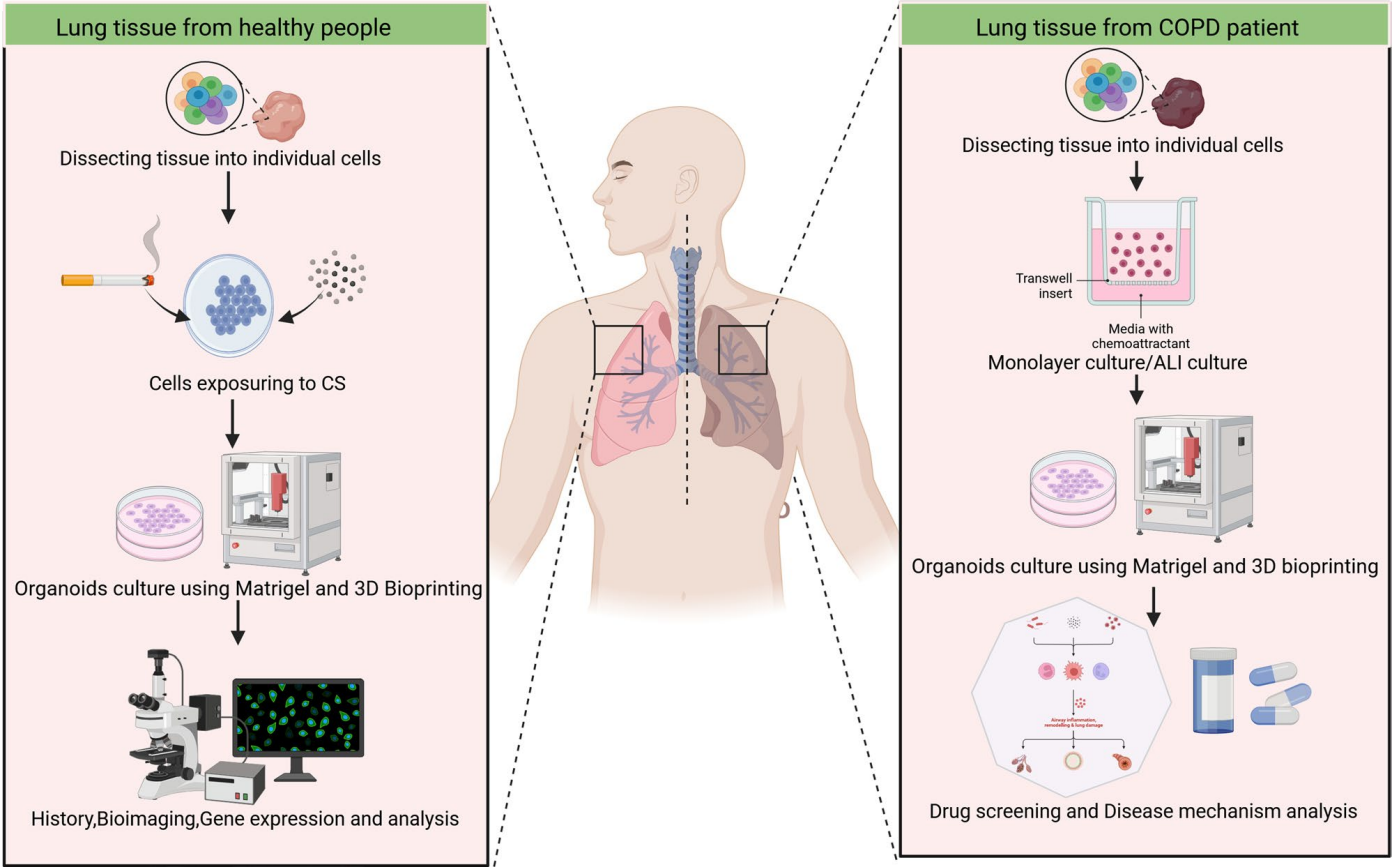
Application of lung organoids in COPD. There are two principal methods for establishing organoids of a COPD model. One method involves exposing healthy cells to CSE or PM2.5, while the other entails the use of cells derived from COPD patients. Subsequently, the cells are cultivated to form three-dimensional organoids, employing Matrigel and three-dimensional bioprinting. Lung organoids of COPD can be employed to elucidate the mechanisms underlying disease onset and related gene expression, as well as to identify targeted drugs that may contribute to precision medicine

The use of lung organoids in the study of the mechanisms underlying COPD is a promising avenue of research(Fig. 3). Two principal methodologies exist for the establishment of organoid models of COPD. One method involves exposing healthy human stem cells to cigarette smoke extract (CSE) or a specific substance (PM2.5), while the other employs the use of nasopharyngeal and bronchial epithelial cells obtained directly from COPD patients to construct organoid models [57–59](Table 1).

The development of nasopharyngeal and bronchiolar organoids using both healthy people and COPD patients revealed the presence of goblet cell hyperplasia and a reduction in ciliary beat frequency in the COPD organoids, in comparison to the healthy organoids [57]. In COPD patient-derived alveolar organoids, respiratory airway secretory (RAS) cell populations display altered transcriptome profiles, which in turn result in altered AT2 cells. Furthermore, the conversion of RAS cells to AT2 cells appears to be affected by smoke damage, as evidenced by an increase in SCGB3A2 + and LAMP3 + cell populations in a pack-year dependent manner [60]. In 3D organogenesis experiments, evaluation of GFP + AT2 cells demonstrated the enhanced adaptability of AT2 cells to CSE, accelerated proliferation and differentiation by activating stem cell function and augmented anti-apoptotic capacity during the development of COPD. This discovery offers a novel perspective for the study of lung tissue self-repair [61]. Researchers have discovered that CSE down-regulated FZD4 expression in mice, and further, the researchers used AT2 cells isolated from patients with COPD to generate organoids. The treatment of these organoids with FZD4 antagonists resulted in a notable reduction in epithelial cell proliferation mediated by the Wnt/β-catenin signalling pathway, thereby interfering with the normal formation process of the organoids [62]. In mouse lung organoids, studies have shown that PM2.5 exposure significantly diminishes the conversion of AT2 to AT1 cells, thereby contributing to the pathogenesis of COPD [59]. This discovery provides an important clue for revealing the pathogenesis of COPD. The current evidence suggests that elevated WNT-5 A/B expression may contribute to the disease process in COPD. Experiments utilizing a lung organoid model have demonstrated that WNT-5 A and WNT-5B inhibit the growth of lung epithelial progenitor cells, with WNT-5B exhibiting a particularly pronounced effect on alveolar epithelial progenitor cells [63]. This indicates that WNT-5 A/5B signalling influences alveolar repair and may result in the impairment of alveolar repair in patients with COPD. Additionally, it has been demonstrated that transforming growth factor beta (TGF-β) inhibits the transformation of fibroblasts into epithelial cells in lung organoids. This suggests that sustained activation of TGF-β may impede epithelial repair in chronic lung diseases such as COPD. Consequently, the inhibition of TGF-β signalling in mesenchymal stem cells may facilitate epithelial regeneration in COPD patients, thereby alleviating the disease and improving the quality of life of patients [64]. Moreover, in an organoid model formed by co-culture of epithelial cells and fibroblasts, a significant reduction in the total number of organoids and an abnormal increase in the mean diameter of existing organoids were observed when the activation ligand LL-37 or HMGB1 of the receptor for advanced glycosylation end products (RAGE) was introduced. These findings strongly suggest the potential involvement of the RAGE pathway in the pathogenesis of emphysema, thereby opening up a new avenue of research into the pathophysiological mechanisms of emphysema [65]. Interleukin 1b(IL-1b) is a pivotal inflammatory mediator that is elevated during COPD exacerbations. In the organoid model formed by co-culture of lung epithelial cells and fibroblasts, the addition of IL-1b was observed to promote the growth of organoids. Subsequent studies revealed that this effect was reversed to inhibition in organoids formed by fibroblasts that had been pretreated with IL-1b [66]. The data indicate that IL-1b alters the phenotype of fibroblasts, promoting a pro-inflammatory response and shifting their supportive function towards an inhibitory role for epithelial progenitor cells. The findings suggest that the role of chronic inflammation is to facilitate an inhibitory repair response, which in turn contributes to the development of COPD.

A cell population not present in mouse lungs was identified in the human terminal and respiratory bronchioles: the SCGB 3A2 cell population. The scRNA-Seq study revealed that these cells are enriched in individuals with COPD [60, 67]. In an in vitro experimental system utilizing lung organoid culture, the researchers observed that AT2 cells were able to transform into SCGB 3A2 + cells under suitable culture conditions. Moreover, studies have also demonstrated that in lung organoids derived from COPD patients, there is a phenomenon of retransformation of AT2 cells into SCGB 3A2 + cells. This phenomenon directly correlates with in vitro experimental results and clinicopathological status, thereby reinforcing the role of SCGB 3A2 + cells in the pathogenesis of COPD [68]. The Family with Sequence Similarity 13 Member A (FAM13A) gene has been consistently associated with COPD by in genome-wide association studies (GWAS) [69]. Further research indicates that in alveolar organoids exposure to chronic cigarette smoke results in lung epithelial cells lacking FAM13A exhibiting abnormal biological characteristics, specifically manifesting as significantly enhanced proliferation and differentiation. These results suggest that FAM13A dysfunction may play an important role in the pathogenesis of COPD by promoting abnormal cell proliferation and differentiation [70].

The studies mentioned above illustrate the substantial contribution of organoids to the field of COPD research. Lung organoids exhibit a high degree of structural fidelity, comprising cell types derived from multiple germ layers and lineages. This similarity enables organoids to accurately replicate the complex series of changes that occur in COPD patients following prolonged exposure to environmental factors like cigarette smoke. These changes include alterations in cell-cell interactions, cytokine and protein expression, and the activation of signalling pathways. Furthermore, the manipulation of the development of lung organoids derived from COPD patients allows for the exploration of potential strategies to reverse or mitigate the pathological processes in damaged lung tissue.

### Lung organoids for personalized medicine in COPD

In recent years, lung organoids have become a valuable tool in clinical drug screening and lung tissue regeneration therapy. Currently, there is no pharmacological intervention that specifically targets impaired tissue repair in COPD. Song et al. evaluated the therapeutic effect of D-dopachrome tautomerase (DDT) in human and mouse alveolar pulmonary organoid models of COPD, demonstrating a promotion of organoid growth after following DDT administration. They thus propose that DDT serve as a foundation for the advancement of COPD treatment strategies, while also underscoring the necessity for further investigation into the compound’s potential for tumorigenicity [71]. In alveolar organoids formed after exposure to cigarette smoke, receptor agonists of both the prostaglandin receptor (EP) and the prostacyclin receptor (IP) were observed to significantly enhance epithelial repair responses. This finding provides compelling evidence that EP and IP serve as novel and promising drug therapeutic targets during lung repair in COPD [72]. Furthermore, previous studies have demonstrated that the administration of retinoic acid can facilitate lung regeneration in adult rodent models of chronic lung disease. However, clinical trials of retinoic acid in patients with COPD have failed [73, 74]. This suggests that further exploration of the complexity and specificity of retinoic acid signalling in regulating the function of adult lung epithelial progenitor cells is required. John et al. addressed this knowledge gap using an adult lung organoid model. The study demonstrates that the combination of histone deacetylase inhibitors and retinoic acid may represent a novel therapeutic strategy for the treatment of COPD [75]. In a separate study, Costa et al. demonstrated that amlexanox facilitates the formation of lung organoids. In addition, Furthermore, the prophylactic administration of amlexanox was observed to markedly enhance lung function and structure in mice with COPD. Consequently, amlexanox has been identified as a novel potential treatment for COPD [76]. The development of novel therapeutic strategies targeting airway goblet cell metaplasia has the potential to alleviate the symptoms associated with COPD. Studies have shown a reduction in LKB1 expression in the lungs of patients with COPD. The lung organoid culture demonstrated that the deletion of LKB1 in mouse airway (club) progenitor cells facilitated the differentiation of airway goblet cells and the aggregation of pulmonary macrophages [77]. Therefore, LKB1 agonists may be a potential treatment option for COPD.

In general, lung organoid technology has demonstrated a pivotal role in the field of drug screening for COPD. This pioneering technology not only markedly accelerates the pace of drug development, but also provides scientists with a exceptionally precise and realistic testing platform, whereby the intricate physiological and pathological milieu of the human lung is faithfully replicated, thereby expediting the identification and validation of novel therapeutic strategies for COPD. The application of lung organoid technology represents a significant advancement in the development of drugs, with the potential to personalize and precision-target treatments for patients with COPD.

### Advantages of lung organoids in COPD

The application of PSC and ASC-derived organoid offers novel insights into cellular processes and molecular signal transduction, which are pivotal for lung growth and the pathophysiological development of lung diseases such as COPD [78]. In previous studies, research of human lung disease was based on cultures of immortalized cell lines and animal models. The anatomical differences between commonly used laboratory animals, such as mice or rats, and humans lead to a significant lack of functional homology, especially in the respiratory system [79].However, previous researchers have also attempted to develop different COPD disease models. For example, in 2011, Hilaire C. et al. employed an artificial approach to explore the pathogeneses of COPD. They isolated lung epithelial cells from the mouse respiratory tract, and cultured them in a monolayer in an air-liquid interface (ALI) system with cigarette smoke exposure [80]. The ALI culture system permits the cultivation of respiratory epithelial cells that are exposed directly to the air with different stimulations, which more closely resemble real-world changes than the traditional cell culture systems. Nevertheless, the ALI system is not without its limitations. In comparison to lung organoids, the ALI system lacks the tissue structure and microenvironment of a real organ, which is present in in vitro models. This significantly constrains the ability of the ALI system to fully mimic the naive airway epithelium. Moreover, Benam et al. devised a human small airway chip integrated with ALI platform to simulate the exacerbation of COPD. The device comprises an upper air channel and a lower medium channel, separated by a porous PDMS membrane. The membrane is then populated with healthy airway epithelial cells or COPD epithelial cells, with endothelial cells placed in opposition. The upper passage air inflow establishes an ALI environment and stimulates the differentiation of airway epithelial cells [81]. In comparison to lung organoids, this microfluidic model can simulate the effects of cigarette smoke passing through the alveolar-capillary interface. However, it is deficient in other cell types that could also influence the microenvironment. Consequently, it is not suitable for high-throughput screening, and this culture system requires sophisticated technology at a considerable cost.

In general, traditional technologies are often limited in their ability to simulate human disease processes due to species differences, experimental conditions, or model simplification, particularly in comparison with more sophisticated models such as the ALI and chip models. Despite the aforementioned limitations, lung organoid technology offers a promising avenue for more accurately modelling the pathophysiological processes of COPD patients. Moreover, organoids are more patient-specific and can be employed for personalized treatment, as they are invariably derived from the patients’ samples. It is anticipated that lung organoid technology will be integrated and complementary with other traditional technologies in the future. By optimizing the combination of these technologies, a more comprehensive and multi-dimensional COPD research platform can be constructed, which not only facilitates to further analysis of the disease mechanism, but also accelerates the process of drug screening and clinical transformation, and provides more accurate and effective treatment strategies for COPD patients.

### Limitations of lung organoids in COPD

Lung organoids have significant potential for use in COPD studies and applications. However, it is important to consider the limitations of this approach. One limitation of organoids is that they lack an immune system, circulatory system, and ECM. Similarly, lung organoids also exhibit the same deficiencies, as they lack the vascular and immune systems, which play pivotal roles in regulating stem cell behaviour and lung tissue structure formation [82]. Another limitation of lung organoids is that the maturity and controllability of the culture system can only be demonstrated when the culture of repetitive organoids is accurate. It is therefore essential that this consistency is achieved if lung organoids are to be widely applied in various research fields. In particular, under identical experimental conditions, each analogous organoid derived from the same source should exhibit highly comparable characteristics, including size, composition, three-dimensional structure, and gene expression. These challenges are prevalent in numerous research domains. For instance, when organoids are employed in drug screening, it is imperative that the initial culture and stimulation conditions remain consistent to ensure the reliability and reproducibility of the results. However, the size of the organoids is typically disparate, and the efficacy of stimulation or nutrient delivery may fluctuate. Consequently, the ultimate outcomes of experiments may also exhibit variability. A widely recognized medium composition system for the cultivation of lung organoids is still lacking. The discrepancies in these nutrients can result in variations in organoid growth and differentiation, consequently impacting the reproducibility of experimental outcomes [83].

## Conclusions and future prospects

Organoids offer several advantages, including high homology with human organs and tissues, the capacity to maintain original tissue characteristics and genetic consistency over the long term, and a three-dimensional spatial structure formed by different types of epithelial cells. Furthermore, they necessitate a minimal sample size and a brief construction period. They are an efficient in vitro model for lung disease research, drug screening and disease prediction, and meet the ethical requirements for experimentation. Consequently, it is becoming increasingly prevalent in research on lung disease worldwide, particularly in COPD studies. COPD, as a systemic disease, involves the complex interplay of multiple cell types and pathological changes in lung structure, which traditional cell culture models struggle to replicate, particularly the dynamic interactions between cells and the alterations in tissue architecture. Organoids, by forming three-dimensional structures, more accurately simulate the in vivo lung microenvironment and facilitate the study of the interactions between different cell types, thereby overcoming the limitations of two-dimensional culture models. As a result, organoids have become an important tool for investigating the pathogenesis of COPD and other lung diseases.

Organoids, as a novel model with unique advantages, show broad potential for application in COPD research. One area of future research will be the integration of the immune system and circulatory system into the construction of organoids, with the aim of creating models that are more structurally and functionally similar to real organs [84]. The use of chemosynthetic biomaterials can facilitate the provision of adjustable physical stimulation and mechanical tension to organoids, which may prove beneficial in the simulation of disease progression processes in COPD, such as oxidative stress, inflammation, and airway remodeling [85]. A further avenue of enquiry in organoid engineering is the fusion and co-culture of multiple types of organs. The realization of this may necessitate the use of microfluidics and functional biomaterials for the integration of organ systems and highly detailed disease modelling, including COPD [86]. The “multi-organ chip,” which simulates the integrated drug response of the whole body, is poised to become an emerging technology that will enhance the success rate of new drug discovery for COPD and other diseases [87]. Concurrently, the convergence of multiple disciplines, including biomedicine and materials science, and the advent of novel technologies, such as high-throughput sequencing and organ-on-a-chip technology, will accelerate the development of organoids in COPD research. Furthermore, organoids present a potential avenue for artificial lung transplantation, which currently represents the sole viable option for treating chronic lung diseases, including COPD. This process is accompanied by the scarcity of available donor organs, the potential for immune rejection, and the drawbacks of taking immunosuppressive drugs for life. The pace of development is rapid and promising, with the potential for entire lung models capable of being transplanted into humans becoming a reality in the near future. As research progresses, organoids will be able to contribute more effectively to human health in a number of areas, including the understanding of COPD etiology, the development of targeted treatments, the screening of drugs and so on.

**Table 1:** Lung organoids for disease mechanism in COPD

Types of Lung Organoids	Research Purpose	Conclusion	References
Bronchial organoids	Host-pathogen interactions at the individual level in patients with COPD were investigated using in vitro model systems.	Goblet cell hyperplasia and decreased ciliary beat frequency in COPD organoids as opposed to healthy organoids.	[51]
Alveolar organoids	To explore the influence of PM2.5 on COPD.	PM2.5 exposure significantly reduces the transformation of AT2 to AT1 cells, leading to the development of COPD.	[52]
Alveolar organoids	To explore the lineage of progenitor cells in the respiratory tract of human lung and their changes in COPD.	RAS cell populations show altered transcriptome profiles, leading to altered AT2 cell. In addition, the conversion of RAS to AT2 cells appears to be affected by smoke damage.	[53]
Alveolar organoids	To explore the mechanism of lung failure to repair CS-induced damage leading to emphysema.	AT2 cells have superior adaptability to CSE that accelerated proliferation and differentiation by activating stem cell function and enhanced anti-apoptotic ability during the development of COPD.	[54]
Alveolar organoids	To identify and functionally characterize specific FZD receptors that control downstream WNT signaling in impaired lung.	Reduced FZD4 expression in COPD contributes to impaired alveolar repair capacity with reduced Wnt/β-catenin signaling pathway.	[55]
Alveolar organoids	To investigate the effect of WNT-5 A/B on alveolar epithelial progenitor cells in patients with COPD.	WNT-5 A and WNT-5B inhibited the growth of lung epithelial progenitor cells, especially WNT-5B significantly affected alveolar epithelial progenitor cells.	[56]
Bronchial organoids	To explore whether TGF-β-induced fibroblast differentiation into myofibroblasts affects the ability of fibroblasts to support pulmonary epithelial repair in COPD.	Sustained activation of TGF-β may impede epithelial repair in chronic lung diseases such as COPD.	[57]
Alveolar organoids	To explore whether RAGE directly leads to alveolar epithelial injury and abnormal repair response in COPD.	Datas indicate that activation of RAGE by its ligands LL-37 and HMGB1 induces acute lung tissue damage and that this impedes alveolar epithelial repair.	[58]
Alveolar organoids	To investigate how the inflammatory microenvironment affects the involvement of epithelial progenitor cells and their supporting mesenchymal niche cells in distal lung tissue repair in COPD.	IL-1b changes the status of fibroblasts by promoting different inflammatory responses and shifting its supporting function to inhibitory function for epithelial progenitor cells.	[59]
Alveolar organoids	To understand the role of SCGB 3A2 + cells in the pathogenesis of COPD.	In lung organoids from COPD patients, there is a phenomenon of retransformation of AT2 cells into SCGB 3A2 + cells.	[61]
Alveolar organoids	To investigate the mechanism of Fam13A in COPD.	FAM13A dysfunction may play an important role in the pathogenesis of COPD by promoting abnormal cell proliferation and differentiation.	[63]

## Data Availability

No datasets were generated or analysed during the current study.

## Abbreviations

COPD: Chronic obstructive pulmonary disease
ESCs: Embryonic stem cells
iPSCs: Induced pluripotent stem cells
ASCs: Adult stem cells
AT1: Type 1 alveolar epithelium
AT2: Type 2 alveolar epithelium
BASC: Bronchiolar alveolar stem cell
NGFR: Nerve growth factor receptor
IL-13: Interleukin 13
IL-17A: Interleukin 17 A
GCM: Goblet cell metaplasia
FACS: Fluorescence-activated cell sorting
BALOs: Bronchoalveolar pulmonary organoids
MACS: Magnetic bead sorting
hPSCs: human pluripotent stem cells
VAFEC: Ventral foregut endoderm cell
CPM +: Carboxypeptidase M positive
HLOs: Human lung organoids
LBOs: Lung bud organoids
WLO: Whole lung organoids
ABM: Artificial basement membrane
CSE: Cigarette smoke extract
PM2.5: Particular matter 2.5
RAS: Respiratory airway secretory
RAGE: Receptor for advanced glycosylation end products
IL-1b: Interleukin 1b
FAM13A: Family with Sequence Similarity 13 Member A
GWAS: Genome-Wide Association Studies
DDT: D-dopachrome tautomerase
EP: Prostaglandin receptor
IP: Prostacyclin receptor
ALI: Air-liquid interface
ECM: Extracellular matrix
